# Supplementary material for: Monitoring Allosteric Interactions with CXCR4 Using NanoBiT Conjugated Nanobodies
Source: Cell Chem Biol. 2020 Oct 15;27(10):1250–1261.e5. doi: 10.1016/j.chembiol.2020.06.006 (PMC7573392; doi:10.1016/j.chembiol.2020.06.006)
Supplement: Document S1. Figures S1 and S2 [file mmc1.pdf]

Cell Chemical Biology, Volume *27*

Supplemental Information

Monitoring Allosteric Interactions with CXCR4 Using NanoBiT Conjugated Nanobodies

Mark Soave, Raimond Heukers, Barrie Kellam, Jeanette Woolard, Martine J. Smit, Stephen J. Briddon, and Stephen J. Hill

Supplementary Information

Monitoring ligand-induced changes in receptor conformation with NanoBiT conjugated nanobodies

Mark Soave[1,2], Raimond Heukers[3,4], Barrie Kellam[2,5], Jeanette Woolard[1,2], Martine J. Smit[3], Stephen J. Briddon[1,2], Stephen J. Hill[1,2*]

[1]Division of Physiology, Pharmacology and Neuroscience, School of Life Sciences, University of Nottingham, Nottingham, NG7 2UH, UK

[2]Centre of Membrane Proteins and Receptors (COMPARE), University of Birmingham and University of Nottingham, The Midlands, UK

[3]Division of Medicinal Chemistry, Amsterdam Institute for Molecules, Medicines and Systems (AIMMS), VU University of Amsterdam, De Boelelaan 1108, 1081 HZ Amsterdam, The Netherlands

[4]QVQ Holding B.V., Yalelaan 1, 3584 CL Utrecht, The Netherlands

[5]School of Pharmacy, Centre for Biomolecular Sciences, University of Nottingham, Nottingham, NG7 2RD, UK

*Corresponding author: stephen.hill@nottingham.ac.uk (Stephen J. Hill)

Lead author: Professor S J Hill (stephen.hill@nottingham.ac.uk)

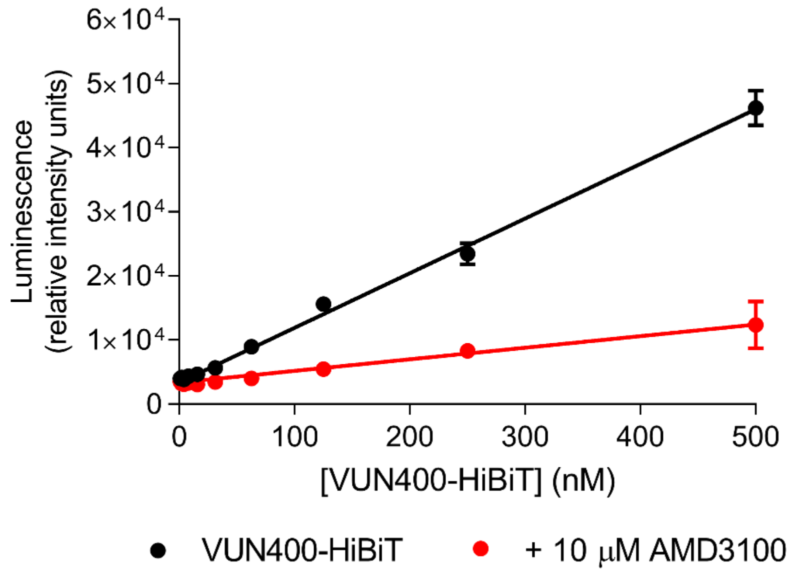

Supplementary Figure 1 (related to Figure 5). Saturation binding of VUN400-HiBiT at the SNAP-CXCR4 in the absence (black circles) or presence of 10 µM AMD3100 (red circles). Data are mean ± SEM from triplicate determinations in a single experiment. This single experiment is representative of five separate experiments.

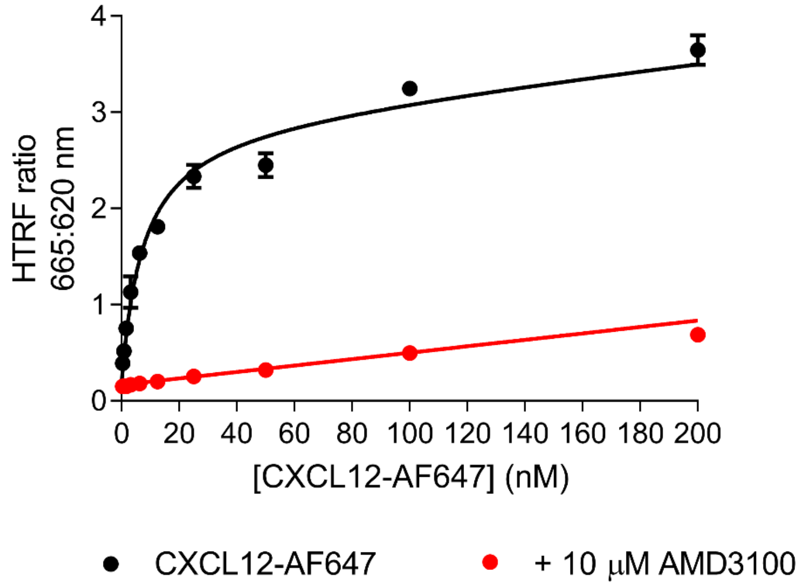

Supplementary Figure 2 (related to Figure 5). TR-FRET saturation binding of CXCL12-AF647 at the SNAP-CXCR4 in the absence (black circles) or presence of 10 µM AMD3100 (red circles). Data are mean ± SEM from triplicate determinations in a single experiment. This single experiment is representative of five separate experiments.
